# Announcing the Andy Kaplan Prize and call for nominations

**DOI:** 10.1186/1742-4690-4-3

**Published:** 2007-01-18

**Authors:** Alice Telesnitsky

**Affiliations:** 1 Department of Microbiology and Immunology, 1150 W. Medical Center Dr. Room 5641, Ann Arbor MI 48109-0620, USA

## Abstract

The retrovirology community has founded a new prize in tribute to our late colleague, Andrew Kaplan, with the goals of honoring and advancing the career of a postdoctoral researcher in our field. We seek help identifying outstanding candidates for this new award.

## Commentary

A new prize in tribute to Andy Kaplan has been established, to be awarded annually, with the purpose of advancing the career and honoring the accomplishments of a distinguished postdoctoral scientist as he or she embarks on an independent career in retrovirology. Until his untimely death at 47 last year, Andy Kaplan was an Associate Professor in Medicine and in Microbiology and Immunology at UNC Chapel Hill [1].

Like many people in the retrovirus field, Andy started attending the Cold Spring Harbor Retrovirus Meeting while he was a postdoc. Many of us got to know Andy and his engaging personality through this meeting, and it was always a pleasure sharing his excitement about science and his interest in junior investigators. The format of the Cold Spring Harbor meeting – predominantly short talks describing the latest results, presented by the people who performed the work themselves – has long highlighted and supported scientists in training. With this new prize, we honor Andy Kaplan's legacy while celebrating the triumphs and challenges of newcomers to our field.

The inaugural Andy Kaplan Prize will be awarded this May at the Cold Spring Harbor Retroviruses meeting. It will consist of a modest cash prize (the exact amount to be determined by the fund advisor) and an opportunity for the recipient to present a short address on his or her research. We anticipate that the award will become an integral part of the Cold Spring Harbor Retroviruses legacy and a valuable laurel for a new investigator in our field.

We ask that you bring this announcement to the attention of eligible applicants. The Kaplan Prize will be awarded to an accomplished young retrovirologist who will have completed his or her PhD no more than four years prior to May 2007. An application for the First Annual Andy Kaplan Prize – which is due February 14, 2007 – will consist of materials similar to those required to apply for a faculty position.

Specific application information is available at a website [2] that can be accessed by Googling "the Andy Kaplan Prize".

Thank you for helping us identify outstanding candidates for this new award, which should serve equally to help advance a new colleague's career and to honor the memory of Andrew Kaplan.

As a final note, the Andy Kaplan Prize is a community effort that will be funded by contributions from those of us in the field. Your modest contribution is most sincerely welcomed and will be crucial to the prize's continuation. The Prize web site [2] lists the financial supporters of this award and describes how you can join this distinguished group.

## Competing interests

The author declares that she is one of the founders of the Andy Kaplan Prize.
